# Theory of mind deficits partly mediate impaired social decision-making in schizophrenia

**DOI:** 10.1186/s12888-017-1313-3

**Published:** 2017-05-05

**Authors:** Liuqing Yang, Peifu Li, Haiying Mao, Huiling Wang, Chang Shu, Vibeke Bliksted, Yuan Zhou

**Affiliations:** 1 CAS Key Laboratory of Behavioral Science, Institute of Psychology, Beijing, 100101 China; 2 Department of Psychology, University of Chinese Academy of Sciences, Beijing, 100049 China; 3 Sino-Danish Center for Education and Research, Beijing, 100190 China; 4 Department of Psychiatry, Renmin Hospital of Wuhan University, Wuhan, 430060 China; 5 Aarhus University Hospital Risskov, Psychosis Research Department, Skovagervej 2, 8240 Risskov, Denmark; 6 Aarhus University, Interacting Minds Centre, Jens Chr. Skous Vej 4, 8000 Aarhus C, Denmark

**Keywords:** Social decision-making, Theory of mind, Neurocognition, Schizophrenia, Mini ultimatum game

## Abstract

**Background:**

Using paradigms from game theory, researchers have reported abnormal decision-making in social context in patients with schizophrenia. However, less is known about the underpinnings of the impairment. This study aimed to test whether theory of mind (ToM) deficits and/or neurocognitive dysfunctions mediate impaired social decision-making in patients with schizophrenia.

**Methods:**

We compared thirty-five patients with schizophrenia to thirty-eight matched healthy controls with regard to social decision-making using the mini Ultimatum Game (mini UG), a paradigm from game theory. Additionally, we assessed ToM using the Theory of Mind Picture Stories Task, a mental state attribution task, and assessed neurocognition using the Brief Assessment of Cognition in Schizophrenia. Mediation analyses were performed on the data.

**Results:**

In contrast to the behavioral pattern of healthy controls in the mini UG, the patients with schizophrenia significantly accepted more disadvantageous offers and rejected more advantageous offers, and showed reduced sensitivity to the fairness-related context changes in the mini UG. Impaired ToM and neurocognition were also found in the patients. Mediation analyses indicated that ToM but not neurocognition partially mediated the group differences on the disadvantageous and advantageous offers in the mini UG.

**Conclusions:**

Patients with schizophrenia exhibited impaired social decision-making. This impairment can be partly explained by their ToM deficits rather than neurocognitive deficits. However, the exact nature of the ToM deficits that mediate impaired social decision-making needs to be identified in future.

**Electronic supplementary material:**

The online version of this article (doi:10.1186/s12888-017-1313-3) contains supplementary material, which is available to authorized users.

## Background

Decision-making is important in daily life and has significant consequences. As human beings, since we live in a complex social world, our decision-making during interaction is not only shaped by our individual goals but also by those of others’ [1, 2]. Decision making in the context of others is termed social decision-making, or more specifically, strategic interactive decision-making. Paradigms from game theory, which have interactive characters, have been increasingly used to investigate social decision-making [1]. Using these simulating games of interpersonal and group interactions, abnormal social decision-making behaviors have been reported in patients with schizophrenia, such as less trust in investment with another counterpart [3], non-strategically less free riding in public goods game [4], and less rejections of unfair offers when splitting a sum of money with a counterpart [5, 6]. However, the underpinnings of these deviant behaviors in schizophrenia are still unclear.

Previous studies have implicated that an ability termed Theory of Mind (ToM) may be a candidate mechanism of social decision-making [1, 7]. ToM refers to the cognitive ability to represent one’s own and others’ mental states to further explain and predict behaviors [8]. This ability is often assumed to be involved in social decision-making, in which inferring intentions of others involved in the task is implicitly required. We can take the ultimatum game (UG) as an example. During this game, two players (proposer and responder) obtain a sum of money together. The proposer first specifies how to split the money between the two of them, and then the responder makes a decision to accept or reject the offer. If the offer is accepted, the two players will get their own share; if it is rejected, neither of them receives anything [9]. Researchers observed that healthy participants as responders showed both behavioral and neural differences between the situations in which, respectively, the proposer is a human with intentions or a computer [10, 11]. Thus, the potential involvement of ToM has been suggested. Specifically, in behavioral studies, rejection rates were higher when the unfair offers were proposed by the human being rather than the computer [11]. Therefore, based on the assumption that it is not necessary to consider the agent’s intention in the computer condition with the other parameters being the same, the differences in the rejection rates reveal the different involvement of ToM in the two conditions. Furthermore, neuroimaging studies found that compared to playing with the computer, playing with the human being produced stronger activation in brain regions which are overlapped with the neural networks of ToM [12], such as the anterior paracingulate cortex and the posterior superior temporal sulcus [10]. Overall, these findings suggest an involvement of ToM in the responder’s behavior in the UG. In contrast to these theoretical predictions and discussion on the potential role of ToM in social decision-making [1, 4, 13, 14], no study has directly measured the relationship between social decision-making and ToM in patients with schizophrenia, in which ToM deficits have been consistently reported [12, 15–19].

The current study aimed to explore the potential relationship between ToM and social decision-making during the mini ultimatum game (mini UG) in patients with schizophrenia. Distinct from the abovementioned classic UG, in the mini UG, the proposer is given two options to choose between on each occasion. One is always 8 vs. 2 (the proposer gets 8 and the responder gets 2) paired with one of four possible alternatives: 5 vs. 5, 2 vs. 8, 8 vs. 2, and 10 vs. 0 [20]. In addition to the chosen option, which corresponds to the proposal in the UG, the additional unchosen alternative in the mini UG can provide clues to the responder to infer intentions underlying the chosen option by the proposer. Consequently, the responder will have different rejection rates to the same chosen option as the alternative changes [20]. Thus, compared to the UG, the modification of the mini UG directly expose the underlying intentions of the proposer’s choices [11, 20–24]. The modification also makes it possible to test whether the unfairness itself or the underlying intentions of the offers drive the responder to make a decision. Therefore, we reasoned that the mini UG should be an efficient paradigm to explore the role of ToM in social decision-making.

In the current study, we first compared the social decision-making behaviors as the responder during the mini-UG in patients with schizophrenia and healthy controls. Next we explored the influence of ToM on responder’s choice during the mini UG. We speculated that compared to the healthy controls, patient with schizophrenia may accept more disadvantageous offers but reject more advantageous offers, based on previous studies, in which the patients with schizophrenia often showed less rejection rates to the unfair (disadvantageous) offers in the classic UG [5, 6] but higher rejection rates to the fair (advantageous) offers in the classic UG [5] or in the mini UG [21]. More importantly, we hypothesized that except in the condition in which the proposer has no alternative (both options are 8 vs. 2), the abnormal behaviors of patients in the mini UG would correlate with their ToM deficits, as they may have difficulty in inferring the intentions of the proposer given the unchosen options. To test this possibility, we used the Theory of Mind Picture Stories Task (TMPST) [25] to measure ToM and examined the mediation effect of ToM in the mini UG. We also noted that neurocognition deficits are well-established in schizophrenia and have a close relationship with social cognition, including ToM [26, 27]. Furthermore, specific aspects of neurocognition, such as working memory and executive function, are closely related to decision-making behaviors [28, 29]. Therefore, in the present study, in addition to the focus on the role of ToM in social decision-making, we also explored the mediation effect of neurocognition on social decision-making.

## Methods

### Participants

Thirty-five patients with schizophrenia, who completed the entire experimental tasks, were recruited from the Department of Psychiatry of the Renmin Hospital of Wuhan University. All the patients were diagnosed with schizophrenia according to ICD-10 criteria by trained psychiatrists (HLW and CS). The patients also met the following inclusion criteria: (1)18–60 years of age, (2) at least 9 years of education, (3) right-handed, and (4) Han Chinese. The exclusion criteria for the patients included (1) diagnosis of drug or alcohol dependency, (2) reported history of a neurological disorder or severe head injury, and (3) presence of other severe physical diseases. All the patients (8 outpatients and 27 inpatients) were clinically stable and all except two were receiving antipsychotic medications. All the medications were converted to their chlorpromazine equivalents according to Gardner et al. [30]. We also enrolled 38 healthy controls by word of mouth and bulletin board postings both in the hospital and nearby communities. The healthy controls, whom we matched to the patients on age, gender and educational level, had the same inclusion and exclusion criteria except that healthy controls would be excluded if they or their first-relatives met any diagnosis of a psychiatric disorder according to the ICD-10 criteria. Each participant provided written informed consent before participation. The Ethics Committee of Renmin Hospital of Wuhan University and the Institutional Review Board of the Institute of Psychology, Chinese Academy of Sciences approved the study.

### Procedures

#### Measurements of clinical symptoms and social functioning

All patients were interviewed with the complete Present State Examination (PSE, ICD-10) by two specialists (PFL and HYM) under the supervision of two psychiatrists (HLW and CS). Patients’ basic clinical and demographic information was documented. To further assess each patient’s clinical symptoms and functional outcome, the Scale for Assessment of Positive Symptoms (SAPS) [31], the Scale for Assessment of Negative Symptoms (SANS) [32], the Global Assessment of Functioning (GAF) scale, and the Personal and Social Performance (PSP) scale were also administered.

#### Neurocognitive measures

To estimate the current IQ of all the participants, the Vocabulary and Block Design subtests from Wechsler Adult Intelligence Scale-IV (WAIS-IV) [33] were administered. In addition, Chinese version of the Brief Assessment of Cognition in Schizophrenia (BACS) [34] was used to assess each participant’s neurocognition. The BACS consists of six subtests estimating verbal memory, working memory, motor speed, attention, verbal fluency and executive function. These primary components of cognition have been reported to be significantly impaired in patients with schizophrenia [35].

#### Theory of mind task

We used the Theory of Mind Picture Stories Task (TMPST) to measure participants’ ToM ability. In the TMPST, participants were explicitly instructed to infer the cognitive mental states (e.g., beliefs and intentions) of characters in the pictures and to understand intended deception, detection of cheating, and cooperation indicated in the stories [25]. Specifically, there were six groups of pictures with three themes: cooperation, deliberate deception and cooperation at the cost of a third party. Each group consisted of four pictures and was coupled with two to five questions. Participants were first required to put the four pictures in the correct order. The time they took to lay the pictures out and whether the order was correct were recorded. After the participants sequenced the pictures in the right order by themselves or the experimenter corrected the wrong order for them, participants needed to answer coupled questions related to the mental states of the characters in the pictures [25]. The questions were designed to evaluate the understanding of first-order belief and false belief, second-order belief and false belief, third-order false belief, reality, reciprocity, deception and cheating detection. For example, one of the questions is “What does the boy in red expect the boy in blue to do?” Before the formal test, participants were introduced to two practice tasks [36]. In the end, three primary measures were yielded: a score for the sequencing component (TMPST-S) reflecting the performances of laying the six groups of pictures in order; a score for the questionnaire component (TMPST-Q) reflecting performances of answering coupled questions and a total score (TMPST-T) that is the addition of the TMPST-S and TMPST-Q. The higher the score, the better ToM ability participants have.

#### Mini ultimatum game

**Fig. 1:**
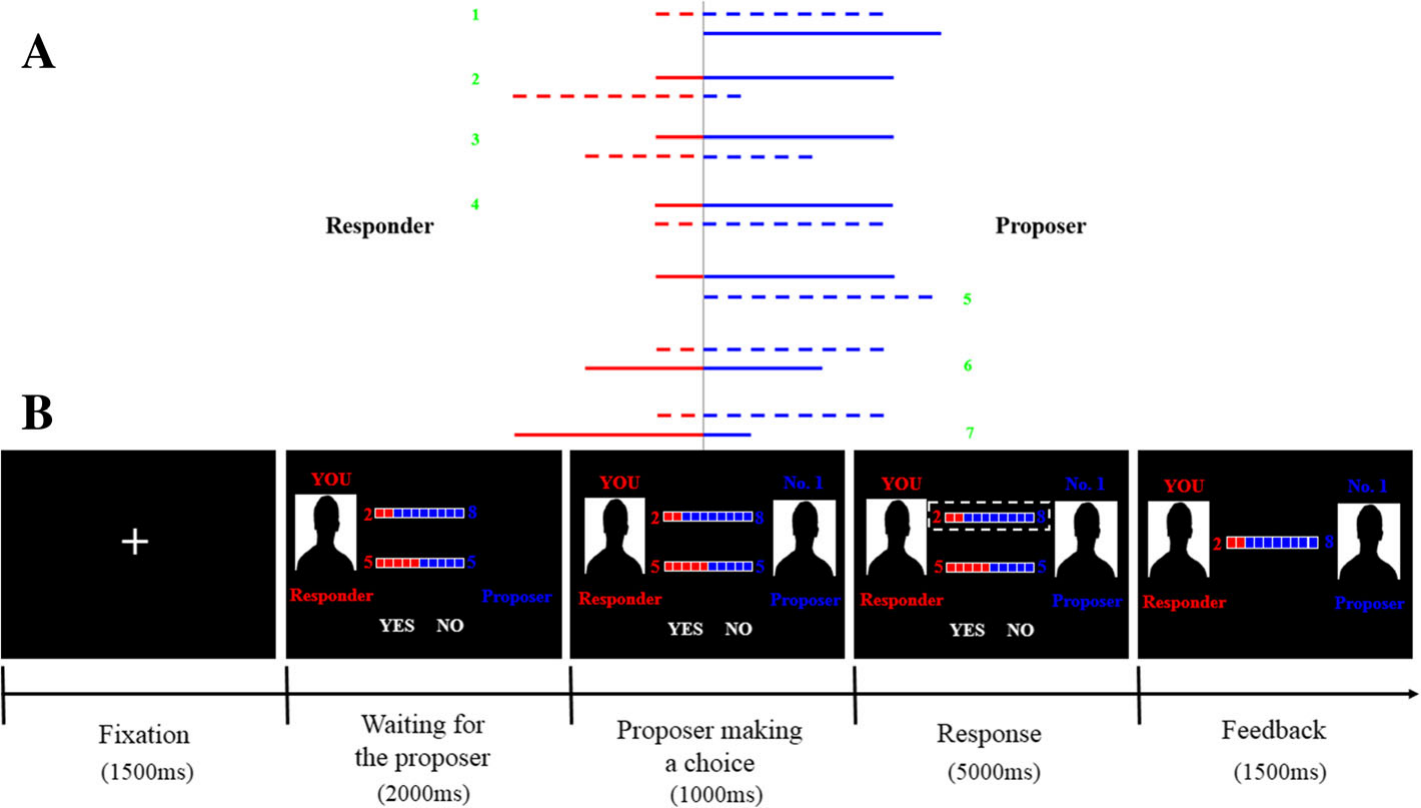
The experimental design of the mini UG. **a** 7 conditions of the proposer’s choices. The blue lines represent the proposer’s own shares and the red lines represent the offers for the responder. The solid lines represent the options chosen by the proposer and the dash lines represent the alternative options. Specifically, the 7 conditions are: 1, hyper-unfair offer (8 vs. 2 / **10 vs. 0**); 2, hyper-fair alternative (**8 vs. 2** / 2 vs. 8); 3, fair alternative (**8 vs. 2 **/ 5 vs. 5); 4, no alternative (**8 vs. 2** / 8 vs. 2); 5, hyper-unfair alternative (**8 vs. 2** / 10 vs. 0); 6, fair offer (8 vs. 2 / **5 vs. 5**); 7, hyper-fair offer (8 vs. 2 / **2 vs. 8**). **b** Diagram illustrating the structure of a single round of the mini Ultimatum Game (mini UG). Each trial started with a 1500 ms fixation interval. In the second stage, picture (*left*) representing the responder, options (here 8 vs. 2 / 5 vs. 5) for the proposer and choices for the responder (YES vs. NO) first appeared, and lasted for 2000 ms. Then the picture (*right*) representing the proposer appeared, and 1000 ms later, the chosen option was encircled in *white*. Participants as responders had at most 5000 ms to make a choice. The feedback of their decision would last for 1500 ms. Here is an example of a trial in the human proposer condition in the mini UG

In the mini UG, the proposer is given two options to choose between on each occasion. One is always 8 vs. 2 (the proposer gets 8 and the responder gets 2), paired with one of the four possible alternatives: fair alternative (5 vs. 5), hyper-fair alternative (2 vs. 8), no-alternative (8 vs. 2), and hyper-unfair alternative (10 vs. 0) [20]. Therefore, there are 7 conditions from disadvantageous to advantageous proposals to the responder: hyper-unfair offer (8 vs. 2 / **10 vs. 0**), hyper-fair alternative (**8 vs. 2** / 2 vs. 8), fair alternative (**8 vs. 2** / 5 vs. 5), no alternative (**8 vs. 2** / 8 vs. 2), hyper-unfair alternative (**8 vs. 2** / 10 vs. 0), fair offer (8 vs. 2 / **5 vs. 5**), and hyper-fair offer (8 vs. 2 / **2 vs. 8**) (the black and bold proposal represents the one chosen by the proposer each time) (Fig. 1a).

The mini UG was administered on the computer, and all the participants played as the responder. They were instructed to play with different persons online or with the computer, but actually it was a programmed version made by the authors (LQY and YZ). Before the formal task, participants were instructed verbally and did a practice task first to make sure that they had understood the game. There were 44 trials in the formal part. Specifically, there were 8 trials for each of the following four conditions, hyper-fair alternative, fair alternative, no alternative and hyper-unfair alternative conditions; and 4 trials were distributed to each of the last three conditions, hyper-unfair offer, fair offer, and hyper-fair offer conditions. Each trial included five phases: fixation, waiting for the proposer, proposer making a choice, response and feedback (Fig. 1b).

### Data analyses

#### Group differences analyses

Most of the data analyses were conducted using SPSS 20.0. Demographic data and the estimated IQ were analyzed using the Mann-Whitney U test or the independent t-test. For the BACS, we first needed to acquire the composite score. We started from standardizing all the participants’ raw scores from each subtest by computing Z-scores relative to the mean and standard deviation of the healthy control group. The composite score (BACS-T) for each participant was attained by averaging all the six subtests’ Z-scores and standardizing the averaged score in the same way mentioned above [35]. With the six subtests scores, the final composite score of BACS and the three measures from the TMPST (questionnaire, sequencing, and total scores), analysis of covariance (ANCOVA) with estimated IQ controlled as a covariate was performed. For the mini UG, we performed repeated measures ANCOVA with estimated IQ as a covariate, taking rejection rates as the dependent variable, the diagnosis (schizophrenia, healthy controls) as a between-subjects factor, and both the proposer type (human, computer) and the proposer’s choices (7 conditions: hyper-unfair offer, hyper-fair alternative, fair alternative, no alternative, hyper-unfair alternative, fair offer, and hyper-fair offer) as within-subjects factors. To explore the sensitivity to fairness-related context in the mini UG, for each participant, we used a binary logistic regression to analyze the effect of the categorical predictor of the seven conditions on the decisions (accept or reject) of participants with mnrfit in MATLAB. In this model,

$$ \mathrm{In}\left(\frac{\pi_{reject}}{\pi_{accept}}\right)={\beta}_0+{\beta}_1\times fairness $$

fairness represents seven fairness conditions (hyper-unfair offer, 0; hyper-fair alternative, 1; fair alternative, 2; no alternative, 3; hyper-unfair alternative, 4; fair offer, 5; hyper-fair offer, 6); π_reject_ and π_accept_ respectively denotes the probability of rejecting or accepting an offer; β_0_ corresponds to the intercept and β_1_ corresponds to the slope which indicates the sensitivity to fairness-related context [37]. After acquiring both the intercept and slope of the regression model for each individual, we separately compared their group differences with independent two-sample *t*-tests.

#### Mediation analyses

**Fig. 2:**
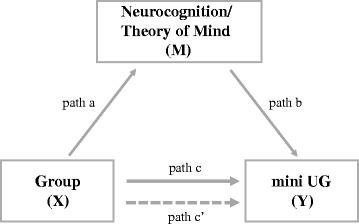
Schematic illustration of hypothetical mediation effects of ToM or neurocognition in the mini UG. Independent variable X influences the dependent variable Y directly (c’) and indirectly (ab) through the mediator M. The direct and indirect effects sum to yield the total effect (c) of X on Y

Mediation analyses were performed to examine the extent to which patients’ abnormal mini UG performances were related to their ToM deficits or neurocognitive dysfunctions. In the classic mediation model, several paths must be estimated (Fig. 2). First, the total effect of the independent variable (X) on the dependent variable (Y) should exist (*c* ≠ 0). Second, the independent variable (X) should significantly predict the mediator (M) (*a* ≠ 0), and the mediator (M) should also significantly predict the dependent variable (Y) with the independent variable (X) controlled (*b* ≠ 0) [38]. Subsequently, researchers have developed new methods with greater statistical power to directly test the indirect effect of X on Y, which was defined as the product of path *a* and path *b* (*ab*) [39].

In this study, we first followed the standard procedure to find potential indices of mediator from the TMPST and BACS measures. To be a mediator, the measures needed to show significant group differences (*a* ≠ 0), and be significantly correlated with the rejection rates in the mini UG on which significant group differences also existed (*b* ≠ 0). The relationships were assessed by partial correlation analyses with the estimated IQ as a covariate. We subsequently used the non-parametric bootstrapping procedure to derive bias-corrected 95% CIs of the *ab* based on 5000 bootstrap samples with estimated IQ controlled. If the CI did not include zero, then *ab* was significantly different from zero (*P* < 0.05), and the conclusion could be drawn that neurocognition or ToM did mediate the group differences in the mini UG.

#### Correlation analyses

In patients with schizophrenia, Spearman rank correlation was used to analyze the relationships between clinical symptoms and behaviors in the mini UG. To explore the potential influences of medication on behaviors of patients with schizophrenia, correlations were analyzed between the chlorpromazine equivalents (CPZ) and the estimated IQ, measures of ToM and neurocognition, and performances in the mini UG.

## Results

### Demographics, estimated IQ and clinical measurements

The demographics and estimated IQ of the two groups, along with patients’ clinical information were shown in the Table 1. There were no significant group differences on any of the demographic variables. The age range of both groups is 18–52. Compared to the healthy controls, the patients with schizophrenia had significantly lower estimated IQ (*P* = 0.003).

**Table 1:** Demographics, estimated IQ and clinical assessments

	SZ (*N*= 35) Mean (SD)	HC (*N* = 38) Mean (SD)	*P*
Gender (male, *N* %)	15 (41.7%)	21 (55.3%)	0.20^a^
Age (in years)	28.46 (7.94)	30.32 (9.15)	0.36^b^
Education (in years)	13.14 (2.74)	13.66 (2.29)	0.39^b^
Estimated IQ	92.71 (18.29)	105.24 (16.95)	0.003^b^
First-episode (*N* %)	9 (22.9%)	-	-
Duration of illness (in months)	60.85 (65.55)	-	-
Times of hospitalization	2.32 (1.80)	-	-
SAPS	13.66 (10.55)	-	-
SANS	33.31 (15.56)	-	-
PSP	53.57 (8.37)	-	-
GAF	54.14 (7.95)	-	-
Medication (CPZ equivalents)^c^	653.47 (726.58)	-	-

*SZ* patients with schizophrenia, *HC* healthy controls, *SD* standard deviation, *SAPS* Scale for the Assessment of Positive Symptoms, *SANS* Scale for the Assessment of Negative Symptoms, *PSP* the Personal and Social Performance scale, *GAF* the Global Assessment of Functioning scale, *CPZ* chlorpromazine

^a^Mann-Whitney U test; ^b^ independent t-test; ^c^ two patients were not receiving antipsychotic medication and were excluded for CPZ calculation

### Group differences in the mini UG

**Fig. 3:**
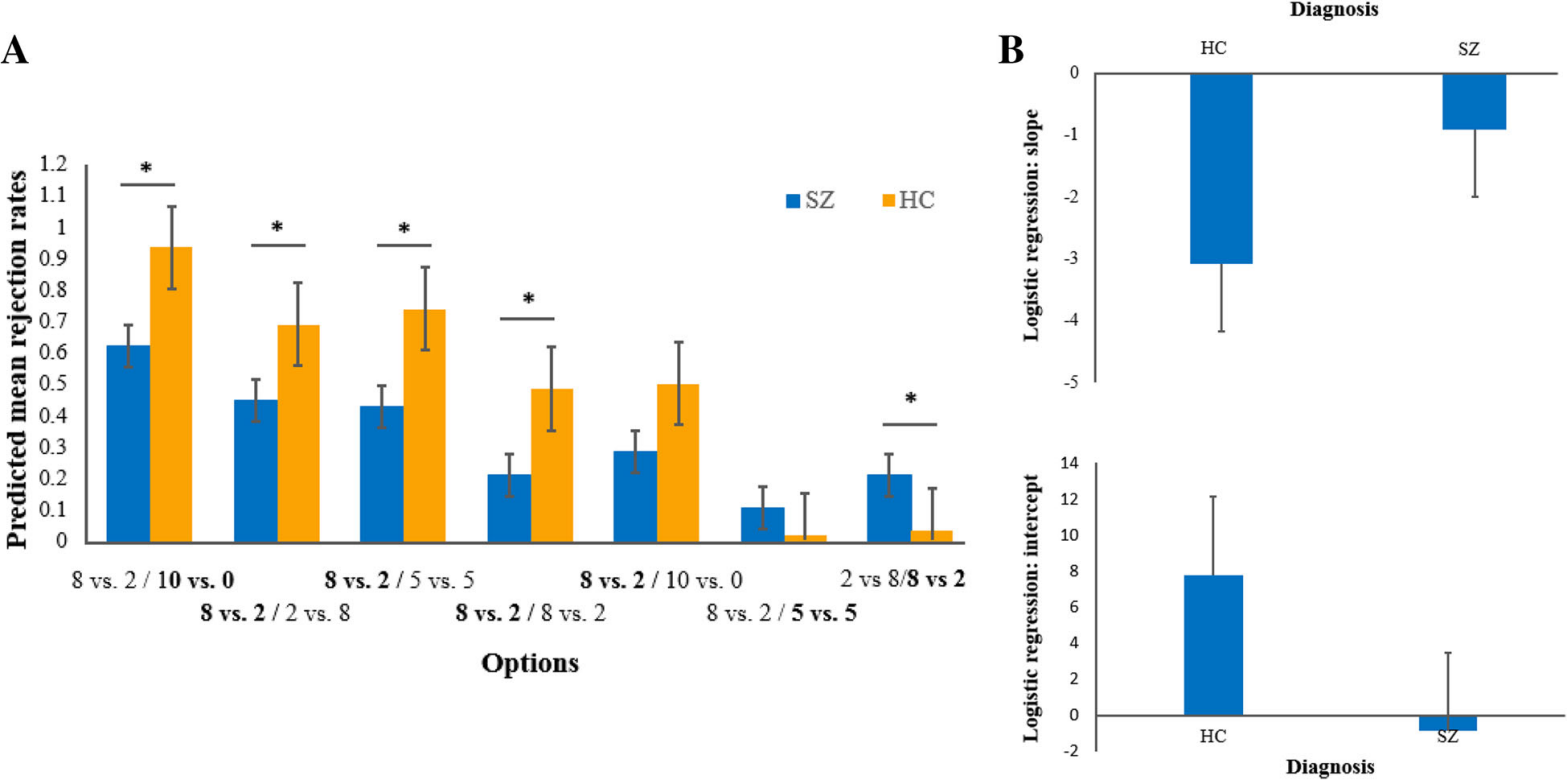
Group differences of performances in the mini UG. **a** Rejection rates in the mini UG with regard to proposer’s choices and group. Error bars indicate standard errors of the mean. Significant group differences are indicated by an asterisk (*, *P* < 0.05). **b** Group differences in the averaged slope and intercept of the binary logistic regression model which represents the effect of the categorical predictor of the seven conditions on the decisions (accept or reject) of participants

In the mini UG, significant main effects of diagnosis (*F*(1,70) = 4.141, *P* = 0.046, partial *η* ^2^ = 0.056), proposer’s choices (*F*(6,65) = 8.660, *P* < 0.001, partial *η* ^2^ = 0.11), and the interaction between them (*F*(6,65) = 5.930, *P* < 0.001, partial *η* ^2^ = 0.078) were found. Simple effect analyses of the interaction effect indicated that in the four disadvantageous conditions, hyper-unfair offer (8 vs. 2 / **10 vs. 0**), hyper-fair alternative (**8 vs. 2** / 2 vs. 8), fair alternative (**8 vs. 2** / 5 vs. 5), and no alternative (**8 vs. 2** / 8 vs. 2), the patients’ rejection rates were significantly lower than those of the healthy controls (all Bonferroni corrected *P* < 0.05). In one of the advantageous condition, i.e., hyper-fair offer (8 vs. 2 / **2 vs. 8**), the patients with schizophrenia showed more rejections (Bonferroni corrected *P* = 0.017) (Fig. 3a). No other significant effects were found.

Figure 3b shows the group differences in the slope and intercept of the binary logistic regression model, which represents the influence of fairness-related contexts on rejection rates. The patients with schizophrenia had significantly decreased absolute values of both the slope and intercept (respectively, *P* = 0.001 and *P* = 0.011). These results suggested that the influence of fairness-related contexts on rejection rates was reduced in the patients with schizophrenia.

### Mediation analyses of the performances during the mini UG

To explore the underpinnings of group differences during the mini UG, mediation analyses were respectively performed to test the mediation effect of ToM and neurocognition (BACS).

#### Mediation effect of ToM

In the TMPST, the healthy control group had significantly higher questionnaire and total scores (both *P* < 0.05) (Table 2). As the sequencing scores showed no significant group differences, the significance in the total scores should be driven mainly by the questionnaire scores. Therefore, in the later mediation analyses, we only took the questionnaire score as the potential index of the mediator. According to the partial correlation analyses, the questionnaire scores significantly related to the rejection rates in the hyper-unfair offer (8 vs. 2 / **10 vs. 0**), fair alternative (**8 vs. 2** / 5 vs. 5), hyper-fair alternative (**8 vs. 2** / 2 vs. 8), and hyper-fair offer (8 vs. 2 / **2 vs. 8**) conditions (respectively, *r* = 0.45, *P* < 0.001; *r* = 0.33, *P* < 0.01; *r* = 0.33, *P* < 0.01; *r* = −0.37, *P* < 0.01). Based on the significant partial correlation results, further bootstrap analyses indicated that in the mini UG, the TMPST questionnaire scores could mediate the group differences in the four above-mentioned conditions (Table 3).

**Table 2:** Group differences in the TMPST and BACS

	SZ (*N* = 35) Mean (SD)	HC (*N* = 38) Mean (SD)	*P* ^a^
TMPST			
Questionnaire scores	16.74 (3.81)	20.37 (3.00)	0.001
Sequencing scores	27.60 (6.84)	30.18 (5.40)	0.319
Total scores	44.40 (9.72)	50.63 (7.31)	0.039
BACS^b^			
Verbal memory	37.34 (11.87)	46.66 (7.85)	0.007
	−1.18 (1.51)		
Digit sequencing	19.00 (4.48)	22.29 (3.50)	0.026
	−0.94 (1.28)		
Token motor	69.03 (15.86)	76.05 (13.93)	0.278
	−0.50 (1.14)		
Verbal fluency	28.46 (10.99)	41.21 (11.20)	*P* < 0.001
	−1.14 (0.98)		
Symbol coding	45.11 (12.19)	61.53 (9.44)	*P* < 0.001
	−1.74 (1.29)		
Tower of London	14.40 (5.61)	16.84 (3.32)	0.225
	−0.74 (1.69)		
Composite score	−1.70 (1.56)		*P* < 0.001

*TMPST* Theory of Mind Picture Stories Task, *SZ* patients with schizophrenia, *HC* healthy controls, *SD* standard deviation.

^a^Significant level of analysis of covariance (ANCOVA) with estimated IQ as a covariant

^b^Both the BACS raw scores and Z-scores of the patients’ group were listed in the table; as the mean Z-score was set to 0 and the standard deviation was set to 1 in the healthy control group, only raw scores were listed

**Table 3:** Mediation effects of ToM and neurocognition in the mini Ultimatum Game

Dependent variables	a	b	c	c’	ab	SE	Effect Size (ab/c)	95% C.I.	
								LL	UL
TMPST-Q									
Hyper-unfair offer (8 vs. 2 / **10 vs. 0**)	2.693	0.034	0.231	0.140^a^	0.091	0.047	39%	0.026	0.211
Fair alternative (**8 vs. 2** / 5 vs. 5)	2.693	0.031	0.218	0.134^a^	0.083	0.049	38%	0.010	0.198
Hyper-fair alternative (**8 vs. 2** / 2 vs. 8)	2.693	0.034	0.211	0.119^a^	0.092	0.052	44%	0.013	0.211
Hyper-fair offer (8 vs. 2 / **2 vs. 8**)	2693	−0.026	−0.172	−0.103^a^	−0.070	0.042	41%	−0.179	-0.011
BACS-T									
Hyper-unfair offer (8 vs. 2 / **10 vs. 0**)	1.214	0.057^a^	0.231	0.162^a^	0.069	0.056	-	−0.036	0.188
Hyper-fair offer (8 vs. 2 / **2 vs. 8**)	1.214	−0.047^a^	−0.172	−0.116^a^	−0.057	0.047	-	−0.187	0.014
TMPST-Q + BACS-T									
Hyper-unfair offer (8 vs. 2 / **10 vs. 0**)	2.693	0.031	0.231	0.119^a^	0.112	0.055	48%	0.005	0.221
Hyper-fair offer (8 vs. 2 / **2 vs. ** **8**)	2.693	−0.023	−0.172	−0.084^a^	−0.089	0.054	52%	−0.214	−0.006

*a*, effect of X on M; *b*, effect of M on Y; *c*, total effect of X on Y; *c’*, direct effect of X on Y; *ab*, mediation effect; *SE*, standard error of estimation of *ab*; *C.I.*, confidence interval of *ab*; TMPST-Q, questionnaire scores from the Theory of Mind Picture Stories Task; BACS-T, composite scores from the Brief Assessment of Cognition in Schizophrenia Scale.

^a^ *P* > 0.05

#### Mediation effect of neurocognition

In the BACS, the patients with schizophrenia performed significantly worse on four out of six subtests and obtained significantly lower composite scores (all *P* < 0.05) (Table 2). Regarding the BACS composite score as another potential mediator, similar mediation analyses were performed. First, we found its significant partial correlations with rejection rates in the hyper-unfair offer (8 vs. 2 / **10 vs. 0**) and hyper-fair offer (8 vs. 2 / **2 vs. 8**) conditions (respectively, *r* = 0.345, *P* = 0.003; *r* = −0.287, *P* = 0.014). However, bootstrap analysis showed that the BACS composite score alone had no mediation effect, unless considered together with the TMPST questionnaire score (Table 3). In addition, considering the potential effects of different aspects of neurocognition on rejection rates in the mini UG, we also analyzed the mediation effects of the four subtest scores on which significant group differences existed. No significant mediation effect was found for each of the subtest scores (Additional file 1: Tables S1 and S2).

### Correlations in patients with schizophrenia

Neither the total scores of the SAPS nor the SANS were significantly correlated with the rejections rates in the mini UG (all *P* > 0.05). Furthermore, in patients with schizophrenia, the CPZ did not relate to estimated IQ, ToM, neurocognition and the mini UG (all *P* > 0.05).

## Discussion

In the current study, we found that the patients showed lower rejection rates to the disadvantageous offers and higher rejection rates to the advantageous offers, and reduced sensitivity to the fairness-related context changes in the mini UG. These findings validated the previous findings [5, 6, 22, 40]. The new contribution of the current work is that we directly investigated the roles of ToM and neurocognition deficits in impaired social decision-making of patients with schizophrenia during the mini UG. We found that the ToM deficits but not dysfunctions in neurocognition, partly mediated the significant group differences in the mini UG during which the intentions of the proposer needed to be inferred.

In the mini UG, an interactive game related to fairness principle in social life, the patients with schizophrenia showed abnormal response patterns compared to the healthy controls. On one hand, patients rejected less disadvantageous offers. Though these offers were in different contexts, all of them were unfair to the participants who played as the responder in this game. This finding echoed the previous observation of less rejections of unfair offers in the classic UG [5, 6] and was also compatible with the finding of more acceptances of unfair offers from the human proposer in populations with schizotypal symptoms [40]. On the other hand, we observed that patients with schizophrenia showed more rejections to the advantageous offers. Higher rejections were found both to the fair and hyper-fair offers, but on the last one group differences achieved a significant level. Still, this finding corroborated the results of previous research using both UG and mini UG that more fair offers were rejected by patients with schizophrenia [5, 22]. In brief, using the mini UG we can detect the abnormal social decision-making behavior in patients with schizophrenia.

Furthermore, we found that the patients showed reduced sensitivity to the change of fairness-related contexts, which suggested that the ToM deficits may be a potential psychopathological mechanism for the altered rejection rates observed in the patients with schizophrenia. In order to directly test the possibility that the ToM deficits may partly explain the abnormal social decision-making in schizophrenia, we analyzed the mediation effect of ToM in the group differences found in the mini UG. We found that the patients with schizophrenia performed worse on the questionnaire scores in the TMPST, suggesting that patients were worse at attributing cognitive mental states (e.g., belief and intentions) of characters in the stories. This finding echoed the consistent observation of ToM deficits in schizophrenia in the previous studies [15, 17, 41]. In conformity with our hypothesis, the questionnaire scores mediated the group differences in rejections both to the disadvantageous and advantageous offers in the conditions when another alternative was available and intentions were exposed, but not in the case when both options were the same 8 vs. 2. Specifically, rejection rates in the three disadvantageous conditions (hyper-unfair offer, hyper-fair alternative, and fair alternative) positively correlated with the TMPST questionnaire scores. The correlations suggested that given the alternative options, the participants with better ToM ability may detect the intentions underlying the choices of the proposer and thus were more likely to reject the intentionally unfair offers. On the other hand, the rejection rates in the advantageous condition (hyper-fair offer) negatively correlated with the TMPST questionnaire scores. This result indicated that the participants with higher ToM abilities appreciated the generous intentions and thus were less likely to reject the hyper-fair offers. Interestingly, Sally and Hill reported that children with autism spectrum disorder (ASD) who had ToM deficits showed similar behavioral pattern in the UG [42], but no other related studies have been found so far. In conclusion, ToM deficits in patients with schizophrenia impair the social strategic ability and make it difficult for the patients to fully integrate intention inferring into their decision-making in the social contexts; thus ToM deficits can partly account for the abnormal social decision-making during the mini UG.

In addition to ToM, we also investigated the role of neurocognition in the social decision-making. Consistent with neurocognition dysfunctions found in schizophrenia [43], the patients in present study acquired significantly less composite scores of BACS. Significant group differences existed on four out of six subtests. In a previous study, researchers suggested that patients with schizophrenia have difficulties in maintaining the representation of the goal and integrating it within the complex context (e.g., the mini UG task) due to their impairments in working memory and proactive control [44]. However, in the present study, we found that the relationships between BACS and performances of patients in the mini UG were weak and mediation analyses showed that the neurocognition (BACS) alone did not have a mediation effect in the group differences. Only considering the working memory measurement (subtest scores of digit sequencing), we also did not find mediation effect. Similar to our case, in a previous study with UG, Csukly et al. also measured participants’ neurocognition including verbal learning and memory, executive control and working memory; they reported that adding these neurocognition measures as covariates into analyses did not affect their results that patients with schizophrenia rejected less unfair offers and more fair offers [5]. No more research was found which directly studied the influences of neurocognition in abnormal social decision-making in patients with schizophrenia. Therefore, more investigations are still needed to further explore the specific relationships between social decision-making and neurocognition. In the present mini UG task, the abnormal behaviors exhibited in the patients with schizophrenia were more related to their impaired ToM rather than their neurocognition deficits.

There are several limitations of this study that need to be addressed. First, in the present study, we did not record the parental level of educational and social economic status in the participants; thus, the influence of parental background on the social decision-making cannot be excluded. Second, the current study did not assess the potential effects of clinical heterogeneity or specific clinical symptoms on social decision-making behavior in patients with schizophrenia, as the relatively small sample size in the current study was inappropriate for categorizing the participants based on their clinical characteristics. Future investigations with subgroups of patients are needed to test whether the abnormal behavioral pattern and its potential mechanism are consistent across these subgroups. For example, it is possible that the decisions made by patients with persecutory delusion may be different from those made by patients with negative symptoms and the underpinnings of social decision-making behavior may also be different, considering that the two subgroups may perform differently on ToM tasks [45–47]. Third, the potential role of emotional ToM ability in social decision-making also requires further exploration, as the task used in the present study focused mainly on the cognitive aspect of ToM. Finally, though the mediation effect of ToM in social decision-making deficits in schizophrenia was identified, the question of what exact aspect of ToM led to the deviant performances in the mini UG cannot be answered based on the current findings. Future studies can address this question using a computational model that can fit behavioral data, i.e., trial-by-trial decisions of patients and healthy controls. Clearly, to parameterize inter-subject variability in a normative or formal sense, a normative model is needed. This could be addressed using the hierarchical Gaussian filter [48] or by active inference for Markov decision processes [49]. The second method may be more suited to the mini UG type of game, and we are currently developing and validating these models for future applications in performance analyses of games, including the mini UG.

## Conclusions

To conclude, patients with schizophrenia exhibited abnormal social decision-making. They rejected less disadvantageous offers and rejected more advantageous offers. They were also not sensitive to the changes of conditions. ToM deficits in patients with schizophrenia impair the social strategic ability and make it difficult for the patients to fully integrate intention inferring into their decision-making in the social contexts. Thus, the patients’ ToM deficits should be one of the underpinnings of their abnormal social decision-making. However, the exact nature of the ToM deficits that mediate this kind of strategic behavior needs to be identified in future.

## Additional file

Additional file 1: Mediation effects of BACS subtests. (DOCX 17 kb)

## Abbreviations

ANCOVA: Analysis of Covariance
BACS: Brief Assessment of Cognition in Schizophrenia.
GAF: Global Assessment of Functioning
PSP: Personal and Social Performance
SANS: Scale for Assessment of Negative Symptoms
SAPS: Scale for Assessment of Positive Symptoms
TMPST: Theory of Mind Picture Stories Task
ToM: Theory of Mind
UG: Ultimatum Game
