# Supplementary material for: Theory of mind deficits partly mediate impaired social decision-making in schizophrenia
Source: BMC Psychiatry. 2017 May 5;17:168. doi: 10.1186/s12888-017-1313-3 (PMC5420154; doi:10.1186/s12888-017-1313-3)
Supplement: Additional file 1: — Mediation effects of BACS subtests. (DOCX 17 kb) [file 12888_2017_1313_MOESM1_ESM.docx]

**Additional file 1**

**Text:**

**Mediation effect of BACS subtests**

Except the composite score of BACS, we also analyzed the mediation effects of the four subtest scores on which significant group differences existed. The results of partial correlations between BACS subtest scores and rejection rates in the mini UG, with IQ controlled, are shown in Table S1. Furthermore, no significant mediation effect was found (Table S2).

**Tables**:

**Table S1. Partial correlations between BACS subtests and mini UG**

|  | Verbal Memory | Digit Sequencing | Verbal Fluency | Symbol Coding |
| --- | --- | --- | --- | --- |
| Hyper-unfair offer  (8 vs. 2 / **10 vs. 0**) | 0.25* | 0.28* | 0.15 | 0.23 |
| Hyper-fair offer  (8 vs. 2 / **2 vs. 8**) | -0.23 | -0.24* | -0.03 | -0.33** |

*Note*: * *P* < 0.05; ** *P* < 0.01

**Table S2. Mediation effect of BACS subtests in the mini UG**

| **Dependent variables** | **a** | **b** | **c** | **c’** | **ab** | **SE** | **Effect Size**  **(ab/c)** | **95% C.I.** | |
| --- | --- | --- | --- | --- | --- | --- | --- | --- | --- |
|  |  |  |  |  |  |  |  | **LL** | **UL** |
| BACS-Verbal Memory |  |  |  |  |  |  |  |  |  |
| Hyper-unfair offer  (8 vs. 2 / **10 vs. 0**) | 0.830 | 0.038^a^ | 0.231 | 0.200 | 0.031 | 0.034 | - | -0.024 | 0.112 |
| BACS-Digit Sequencing |  |  |  |  |  |  |  |  |  |
| Hyper-unfair offer  (8 vs. 2 / **10 vs. 0**) | 0.583 | 0.058^a^ | 0.231 | 0.197 | 0.034 | 0.027 | - | -0.002 | 0.114 |
| Hyper-fair offer  (8 vs. 2 / **2 vs. 8**) | 0.583 | -0.048^a^ | -0.172 | -0.144 | -0.028 | 0.024 | - | -0.113 | 0.001 |
| BACS-Symbol Coding |  |  |  |  |  |  |  |  |  |
| Hyper-fair offer | 1.504 | -0.057^a^ | -0.172 | -0.087^a^ | -0.085 | 0.058 | - | -0.225 | 0.011 |
| (8 vs. 2 / **2 vs. 8**) |  |  |  |  |  |  |  |  |  |

*Note*: **a**, effect of X on M; **b**, effect of M on Y; **c**, total effect of X on Y; **c’**, direct effect of X on Y; **ab**, mediation effect; **SE**, standard error of estimation of **ab**; **C.I.**, confidence interval of **ab**; BACS, Brief Assessment of Cognition in Schizophrenia Scale.

^a^ *P* > 0.05
